# Peak exercise-to-recovery changes in biventricular volumes and function assessed by exercise cardiovascular magnetic resonance

**DOI:** 10.1093/ehjimp/qyag135

**Published:** 2026-08-05

**Authors:** Alexander Schulz, Nicole C Y Deng, Marcelline Lopes, Long Ngo, Kathryn Arcand, Warren J Manning, Reza Nezafat

**Affiliations:** Department of Medicine (Cardiovascular Division), Beth Israel Deaconess Medical Center and Harvard Medical School, 330 Brookline Ave., Boston, MA 02215, USA; Department of Medicine (Cardiovascular Division), Beth Israel Deaconess Medical Center and Harvard Medical School, 330 Brookline Ave., Boston, MA 02215, USA; Department of Medicine (Cardiovascular Division), Beth Israel Deaconess Medical Center and Harvard Medical School, 330 Brookline Ave., Boston, MA 02215, USA; Department of Medicine (Cardiovascular Division), Beth Israel Deaconess Medical Center and Harvard Medical School, 330 Brookline Ave., Boston, MA 02215, USA; Department of Medicine (Cardiovascular Division), Beth Israel Deaconess Medical Center and Harvard Medical School, 330 Brookline Ave., Boston, MA 02215, USA; Department of Medicine (Cardiovascular Division), Beth Israel Deaconess Medical Center and Harvard Medical School, 330 Brookline Ave., Boston, MA 02215, USA; Department of Radiology, Beth Israel Deaconess Medical Center and Harvard Medical School, 330 Brookline Ave., Boston, MA 02215, USA; Department of Medicine (Cardiovascular Division), Beth Israel Deaconess Medical Center and Harvard Medical School, 330 Brookline Ave., Boston, MA 02215, USA

**Keywords:** exercise CMR, left ventricular function, right ventricular function, biventricular volumes, post-exercise recovery

## Abstract

**Aims:**

Exercise cardiovascular magnetic resonance (Ex-CMR) assesses cardiac function under physiological stress. Imaging during continuous in-bore exercise reflects peak physiology but is challenging, prone to motion artefacts and patient discomfort. Exercise performed outside the scanner followed by rapid post-exercise imaging improves feasibility and image quality, but the time course of ventricular volume recovery after exercise cessation remains poorly defined. We compared biventricular Ex-CMR measurements during steady-state peak exercise with those acquired sequentially after exercise termination.

**Methods and results:**

Ten healthy adults [24 (21–31) years; 8 female] underwent Ex-CMR on a 3T system. Biventricular volumes were assessed using a free-breathing, electrocardiogram-triggered, highly accelerated multi-slice cine sequence with compressed sensing and deep learning-based reconstruction. Short-axis stacks were acquired at rest, during two steady-state peak exercise acquisitions, and at three sequential post-exercise timepoints, with the first recovery scan beginning 10–15 s after cessation. Linear mixed-effects models evaluated recovery categorically and as a function of elapsed time. Differences between repeated peak acquisitions were small [left ventricular (LV) end-diastolic volume +1 ± 3 mL; LV end-systolic volume −4 ± 7 mL]. Immediately after cessation (13 ± 2 s), biventricular volumes remained unchanged vs. peak (all *P* = 1.00). Significant recovery-related changes emerged by 54 ± 9 s and were present for all parameters by 99 ± 18 s. Linear modelling showed LV stroke volume declining 2.1 mL (−1.9%) per 10 s, with similar right-ventricular trends. Post-exercise acquisitions showed higher diagnostic quality and fewer artefacts than in-bore imaging.

**Conclusion:**

Biventricular volumes measured immediately after exercise closely approximate peak physiology, whereas recovery changes occur rapidly and linearly thereafter. Rapid post-exercise acquisition offers a practical surrogate for peak measurements while improving image quality.

## Graphical Abstract

**Graphical Abstract:**
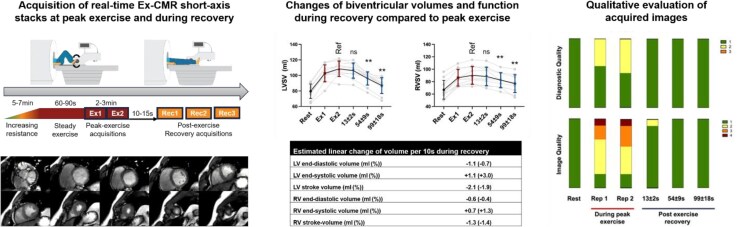
Ex-CMR, exercise cardiovascular magnetic resonance imaging; LV, left ventricle; RV, right ventricle.

## Introduction

Exercise testing is central to cardiovascular medicine, revealing symptoms and haemodynamic abnormalities not apparent at rest. Cardiovascular magnetic resonance (CMR) is the reference standard for quantifying biventricular volumes and function.^1^ Recent advances in image acceleration techniques allow reproducible assessment of biventricular function during physiological exercise.^2,3^ As a result, exercise CMR (Ex-CMR) permits comprehensive functional and haemodynamic characterization.^4^

Supine bicycle ergometry enables imaging during sustained in-bore peak exercise or immediately after termination. While simultaneous in-bore imaging most closely reflects peak physiology, it remains technically demanding due to motion, limited monitoring, patient burden, and bore-related physical constraints that may restrict exercise intensity.

Alternatively, exercise can be performed immediately outside the bore followed by rapid repositioning for imaging. Table-mounted ergometers have shortened bore transfer times and allow near-immediate post-exercise acquisition.^4^ However, although augmentation of cardiac volumes during exercise has been described,^2,4,5^ considerably less is known about the time course of recovery after exercise cessation. It remains uncertain how long cardiac volumes continue to reflect peak stress physiology once exercise has stopped. Importantly heart rate and ventricular volumes may normalize at different rates. Accordingly, whether post-exercise Ex-CMR volumetric assessment serves as an accurate surrogate of cardiac physiology at peak stress or may confound clinical interpretation remains a subject of ongoing debate. This study systematically compared biventricular Ex-CMR volumes acquired during steady-state in-bore exercise with those obtained immediately and sequentially after exercise termination to define recovery-related changes relative to peak physiology.

## Methods

### Participants

The study was approved by the local institutional review board, and all participants provided written informed consent. Self-reported healthy adults were prospectively recruited to undergo Ex-CMR. Exclusion criteria included age <18 years, pregnancy, and height >175 cm, as greater body height may limit the ability to perform unrestricted in-bore bicycle exercise due to bore size constraints. Demographic and anthropometric data were collected, including height, weight, total leg length (anterior superior iliac spine to floor), and upper leg length (tibial plateau to iliac spine).

### CMR imaging protocol

Ex-CMR was performed on a 3T system (MAGNETOM Vida, Siemens Healthineers AG, Forchheim, Germany) with a 75 cm bore. Biventricular volumes and function were assessed using a free-breathing, prospectively electrocardiogram (ECG)-triggered, multi-slice double-beat cine sequence reconstructed at following spatial resolution: 1.9 × 1.9 × 8 mm^3^; temporal resolution 38 ms; TE 1.2 ms; TR 2.8 ms; flip angle 26°. Compressed sensing with non-uniform k-space undersampling was applied. The sequence continuously acquires ECG-triggered cine data for each slice. A combination of compressed sensing and a deep learning-based resolution-enhancement generative adversarial model enables 14.8-fold acceleration.^6^

**Figure 1:**
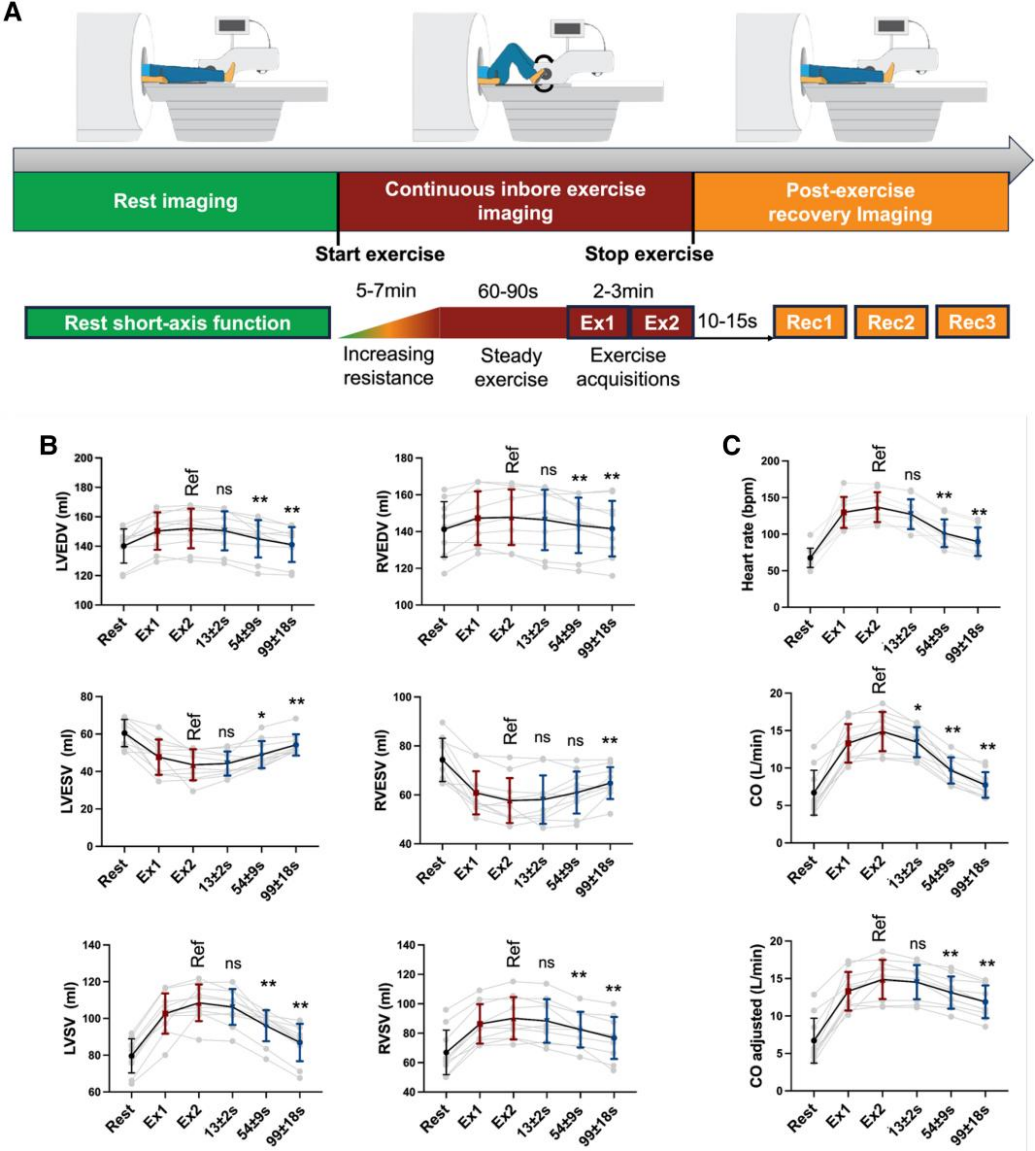
Exercise protocol and measurements of biventricular volumes and cardiac output. (*A*) Schematic of the exercise protocol beginning with resting imaging, followed by continuous in-bore exercise using a ramp protocol until the predefined peak exercise level is reached. This peak workload is maintained for 60–90 s, during which two consecutive exercise acquisitions are obtained (Ex1 and Ex2). Exercise is then terminated, and after a delay of ∼10–15 s, recovery imaging is initiated. In total, three recovery acquisitions are performed (Rec1–Rec3). Each acquisition takes about 30–40 s, depending on the heart rate. (*B*) Dynamic changes in LV and RV EDV, ESV, and stroke volume (SV) measured at rest, during two consecutive acquisitions obtained during continuous in-bore exercise (Ex1 and Ex2), and during three recovery acquisitions (time indicated in seconds after exercise termination). (*C*) Dynamic changes in heart rate, cardiac output (CO), and heart rate-adjusted CO (calculated using peak exercise heart rate rather than the heart rate at the time of acquisition). Measurements were obtained at rest, during Ex1 and Ex2, and during three recovery acquisitions. Values from the second exercise acquisition (Ex2) served as the reference (Ref) for all recovery comparisons. Statistical comparisons vs. the reference scan were performed using linear mixed-effects models with *post hoc* Bonferroni correction: ns, not significant; *P < 0.05; **P < 0.01.

Short-axis stacks covering the ventricles from base to apex were acquired at six timepoints (*Figure 1A*): one at rest, two during continuous peak in-bore exercise, and three following exercise termination. After completion of in-bore exercise with simultaneous functional imaging, patients stopped exercising, and the first recovery acquisition was initiated after 10–15 s, approximating the typical transition time required for out-of-bore exercise followed by post-exercise imaging. Subsequent scans were initiated immediately after completion of the preceding acquisition. Each short-axis stack required ∼30–40 s, depending on heart rate. The exact timing of each acquisition following exercise completion was manually recorded.

### Exercise protocol

*Figure 1* illustrates the exercise protocol. Exercise was performed using a CMR-compatible supine bicycle ergometer (Lode, Groningen, The Netherlands). Resistance was increased approximately every 30–60 s while maintaining cadence, guided by perceived exertion (‘easy,’ ‘moderate,’ and ‘hard’), targeting a sustainable ‘hard’ level for 2–5 min and a total exercise duration of 10–12 min. After reaching peak workload, exercise was maintained for 60–90 s to ensure steady-state physiology prior to image acquisition.

### Image analysis

**Figure 2:**
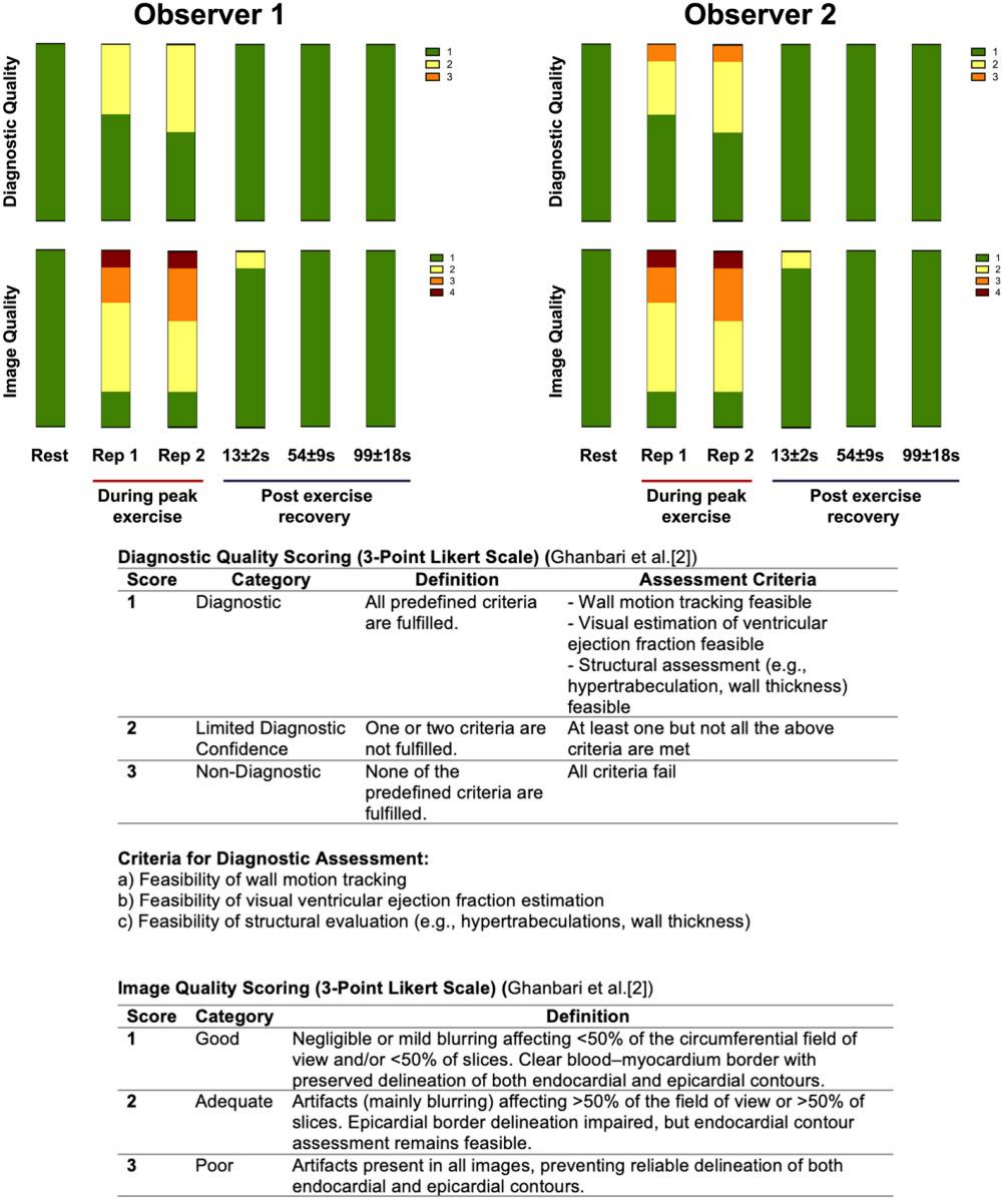
Qualitative assessment of Ex-CMR acquisitions. Diagnostic quality and overall image quality were evaluated according to the predefined scoring systems detailed in the two tables. Assessments were performed by two independent observers at rest, during two consecutive acquisitions obtained under continuous in-bore exercise (Ex1 and Ex2), and during three recovery acquisitions (post-exercise), with recovery time specified in seconds after exercise termination.

Ventricular volumes and function were derived from the short-axis cine stack using cvi42 (version 6.0.2, Circle Cardiovascular Imaging Inc., Calgary, Alberta, Canada), following previously validated protocols.^2^ Details on criteria used for qualitative assessments are shown in *Figure 2*. Endocardial and epicardial borders were automatically detected and manually adjusted as needed by a blinded Level III SCMR trained physician (A.S. 10 years of experience). Qualitative assessments were performed by A.S. and N.C.Y.D. (CMR technologist with one year of experience).

### Statistical analysis

Continuous variables are presented as mean ± standard deviation or median with interquartile range unless otherwise specified. Categorical variables are presented as counts and percentages. Linear mixed-effects models with subject-specific random intercepts accounted for repeated measures. Recovery was analysed categorically (timepoint with Bonferroni-adjusted pairwise comparisons) and continuously (elapsed seconds since exercise termination) to estimate linear change over time. Degrees of freedom were approximated using the Kenward–Roger method (categorical models) and Satterthwaite approximation (continuous models). A two-sided *P* < 0.05 was considered significant. Analyses were performed using R Studio (version 4.4.3) and GraphPad Prism (Version 10, GraphPad Software, San Diego, CA, USA).

## Results

Ten healthy subjects [24 (21–31) years; 8 females] were enrolled. Mean height was 165 cm (range: 155–175 cm), weight 68 kg (range: 58–90 kg), and BMI 24 (22–28) kg/m^2^. Mean hip-to-floor leg length was 95 cm (range: 85–107 cm) and upper leg length 49 cm (range: 43–58 cm). Baseline resting ventricular volumes and function are summarized in *Table 1*. Exercise performance is detailed in *Table 1*. All participants rated peak exertion as ‘hard’.

**Table 1:** Baseline characteristics and exercise performance, and functional recovery after exercise

Parameter				
Age, years				24 (21–31)
Sex female, *n* (%)				8 (80)
Body mass index (kg/m^2^)				24 (22–28)
LV mass index (g/m^2^)				43 (40–49)
LV EDV index (mL/m^2^)				80 (75–85)
LV SV index (mL/m^2^)				44 (42–48)
LV ejection fraction (%)				55 (54–60)
RV EDV index (mL/m^2^)				78 (76–82)
RV SV index (mL/m^2^)				36 (32–37)
RV ejection fraction (%)				46 (42–51)
**Exercise performance and haemodynamic response**				
Maximum resistance (W)				75 (49–90)
Exercise duration (min)				11 (8–12)
Absolute heart rate increase (bpm)				61 (55–89)
Relative heart rate increase (%)				100 (83–132)
Percent of age predicted maximum heart rate (%)				74 (63–78)
Systolic blood pressure rest (mmHg)				106 (99–118)
Diastolic blood pressure rest (mmHg)				68 (60–82)
Systolic blood pressure peak exercise (mmHg)				129 (114–144)
Diastolic blood pressure peak exercise (mmHg)				76 (55–87)

Volume changes during recovery compared with peak exercise				
Cardiac volume	Estimated linear change per 10 s	13 ± 2 s	54 ± 9 s	99 ± 18 s
LV EDV (mL)	−1.1	−1.6 (−4.6 to 1.4)	−7.0 (−10.0 to −4.0)	−10.9 (−13.9 to −8.0)
LV EDV (%)	−0.7	−1.0 (−2.9 to 0.8)	−4.6 (−6.4 to −2.8)	−7.1 (−8.9 to −5.3)
LV ESV (mL)	+1.1	+0.7 (−2.5 to 3.9)	+5.4 (2.2 to 8.6)	+10.6 (7.4 to 13.8)
LV ESV (%)	+3.0	+2.6 (−6.8 to 12.0)	+14.5 (5.1 to 23.9)	+27.6 (18.2 to 37.0)
LV SV (mL)	−2.1	−2.3 (−5.7 to 1.2)	−12.5 (−15.9 to −9.0)	−21.6 (−25.0 to −18.1)
LV SV (%)	−1.9	−2.1 (−5.0 to 0.9)	−11.3 (−14.3 to −8.3)	−19.8 (−22.8 to −16.9)
RV EDV (mL)	−0.6	−1.4 (−3.5 to 0.6)	−4.4 (−6.5 to −2.4)	−6.2 (−8.3 to −4.1)
RV EDV (%)	−0.4	−1.1 (−2.5 to 0.4)	−3.0 (−4.4 to −1.6)	−4.2 (−5.6 to −2.8)
RV ESV (mL)	+0.7	+0.4 (−2.6 to 3.4)	+3.3 (0.2 to 6.3)	+7.2 (4.1 to 10.2)
RV ESV (%)	+1.3	+0.7 (−4.9 to 6.4)	+6.4 (0.8 to 12.0)	+13.6 (7.9 to 19.2)
RV SV (mL)	−1.3	−1.8 (−4.3 to 0.7)	−7.7 (−10.2 to −5.2)	−13.3 (−15.8 to −10.8)
RV SV (%)	−1.4	−2.1 (−5.0 to 0.8)	−8.2 (−11.1 to −5.3)	−15.0 (−17.9 to −12.2)

Volume changes compared with resting acquisition				
Cardiac volume	13 ± 2 s	54 ± 9 s		99 ± 18 s
LV EDV (%)	6.5 (5.8 to 9.2)	3.1 (1.4 to 5.7)		0.7 (−0.2 to 2.1)
LV ESV (%)	−25.9 (−33.5 to −23.2)	−20.1 (−23.0 to −13.5)		−11.4 (−16.8 to −1.3)
LV SV (%)	32.7 (29.9 to 37.6)	18.0 (16.7 to 29.0)		11.1 (3.1 to 14.6)
RV EDV (%)	3.3 (0.6 to 7.1)	1.1 (−0.9 to 2.0)		−0.5 (−1.0 to 0.4)
RV ESV (%)	−26.3 (−29.1 to −11.7)	−20.3 (−22.5 to −10.5)		−9.6 (−17.9 to −7.2)
RV SV (%)	31.8 (22.6 to 43.5)	25.8 (19.0 to 30.7)		17.1 (9.2 to 23.4)

Bold values indicated passed time post exercise termination. LV, left ventricle; RV, right ventricle; EDV, end-diastolic volume; ESV, end-systolic volume; SV, stroke volume.

Images acquired during exercise more frequently showed artefacts and reduced diagnostic quality, whereas post-exercise acquisitions preserved image quality (*Figure 2*). During repeated continuous in-bore exercise acquisitions, heart rate increased further by 4 (4–8) bpm at time of the second acquisition (*P* = 0.013). Volumetric differences between both acquisitions were comparably small, with changes of 2 (0–3) mL for left ventricular (LV) end-diastolic volume (EDV) (*P* = 0.080), −2 (−5 to 1) mL for LV end-systolic volume (ESV) (*P* = 0.098), 0 (−1 to 2) mL for right ventricular (RV) EDV (*P* = 0.473), and a slightly lower RVESV (−3 (−5 to −1) mL, *P* = 0.006).

Detailed recovery dynamics are illustrated in *Figure 1B* and *Table 1*. Estimated linear changes per 10 s for all parameters are presented in *Table 1*. LV and RV volumes were unchanged immediately after exercise cessation (13 ± 2 s) compared with peak exercise (all *P* = 1.00). At 54 ± 9 s, significant differences were observed in all biventricular volumes (all *P* < 0.02), except for RVESV, which showed a non-significant increase (*P* = 0.349). By 99 ± 18 s, all biventricular volumes were significantly altered compared with peak exercise (all *P* < 0.002). Nonetheless, compared with rest, exercise-induced changes in biventricular volumes remained evident at 54 ± 9 s and, to a lesser extent, at 99 ± 18 s, particularly for end-systolic volume and stroke volume (*Table 1*).

Heart rate remained at 93 ± 5% of peak at the first recovery scan (*P* = 0.057), declining to 74 ± 6 and 65 ± 9% at subsequent scans (both *P* < 0.001) resulting in a significantly decreased cardiac output by −1.4 L/min (−9.0%; *P* = 0.032), −5.2 L/min (−34.6%; *P* < 0.001), and −7.12 L/min (−47.5%; *P* < 0.001), respectively (*Figure 1C*). When corrected for heart rate at peak exercise, cardiac output reductions were attenuated to −0.4 L/min (−2.1%; *P* = 1.00), −1.74 L/min (−11.3%; *P* < 0.001), and −3.0 L/min (−19.8%; *P* < 0.001) (*Figure 1C*).

## Discussion

The principal finding of this study is that Ex-CMR-derived end-diastolic and end-systolic biventricular volumes obtained immediately after exercise closely approximate those measured during continuous peak in-bore exercise, with a predictable and time-dependent decline thereafter. While early post-exercise imaging demonstrated excellent concordance with peak measurements, we observed progressive linear recovery with increasing delay, ranging from 0.4% per 10 s for RVEDV to 3% per 10 s for LVESV. These findings support immediate post-exercise imaging as a practical surrogate for peak exercise ventricular function and volumes, while emphasizing the importance of minimizing acquisition delay. In addition, acquiring images immediately after exercise termination improved image quality by reducing motion artefacts and mitigate practical constraints related to patient height and leg size within the scanner bore, thereby enhancing the feasibility and broader clinical applicability of Ex-CMR.

As Ex-CMR has evolved beyond ischaemia assessment towards comprehensive functional phenotyping in heart failure, cardiomyopathies, athletic remodelling, and congenital heart disease, Ex-CMR has gained increasing interest to enable comprehensive assessment of exercise-induced functional and volumetric changes.^5,7–9^ Since out-of-bore exercise offers greater patient comfort, enables closer physiologic monitoring, and has been shown to provide a low artefact burden with high diagnostic confidence, it may facilitate broader clinical application. While earlier work assessed volumetric recovery from gated imaging on biplane long-axis and a single short-axis positions,^10^ three-dimensional volumetric changes capturing global myocardial function, particularly during real-time, non-gated imaging under free breathing, may be different.

Understanding normalization kinetics is essential when interpreting pathological responses in different populations. Although the differences observed in our study (0.4–3% compared with peak stress) appear modest, especially in light of exercise-induced volumetric changes of up to 27% in healthy subjects of similar age,^11^ and reported intergroup differences of ∼10% or more between patients and healthy controls during exercise,^5,7^ timing remains critical. With increasing delay after exercise termination (e.g. ∼30 s corresponding to an ∼9% change in LVESV), recovery-related shifts may substantially influence intergroup comparisons and potentially attenuate true physiological differences.

Furthermore, patients or individuals with lower exercise capacity or impaired training status may demonstrate slower post-exercise normalization. While prolonged recovery could theoretically preserve stress-induced volumetric alterations for a longer duration, intergroup comparisons across disease populations should aim to minimize acquisition delay to reduce bias introduced by differential recovery speeds. For certain Ex-CMR applications, such as cardiac output assessment,^7^ adjustment for peak heart rate, as demonstrated in this study, may partially mitigate these effects. Recovery patterns may vary according to the study population and exercise protocol. Lower submaximal workloads, different exercise durations, or variations in peak exertion may therefore result in distinct recovery trajectories. However, because heart rate recovery is generally slower in patients than in healthy individuals, our findings likely represent a conservative estimate of post-exercise changes.

### Limitations

In prior studies, participants taller than 175 cm frequently experienced restricted leg movement during in-bore exercise and were therefore excluded to avoid bore-related mechanical limitations. Exercise was performed in the supine position and may not fully reflect upright physiology. Other submaximal exercise protocols including different time of exercise or lower peak exertion may result in different recovery. Post-exercise changes in respiratory patterns and intrathoracic pressures may have contributed to the observed recovery kinetics.

## Conclusion

Ex-CMR biventricular volumes obtained immediately after exercise closely reflect steady-state peak measurements, with progressive and near-linear normalization during recovery. Rapid post-exercise acquisition preserves peak physiological fidelity while improving image quality and enhancing the feasibility of Ex-CMR in routine clinical practice.

## Data Availability

All source data are available from the corresponding author upon reasonable request.

## Lead author biography

Dr Alexander Schulz is a cardiology fellow and is a Research Fellow at Beth Israel Deaconess Medical Center and Harvard Medical School, USA. He specializes in multimodality cardiovascular imaging, with a particular focus on cardiovascular magnetic resonance imaging, and is a certified Senior Instructor in Cardiovascular Magnetic Resonance Imaging and Cardiovascular Computed Tomography. His research focuses on non-invasive imaging in hypertrophic cardiomyopathy, heart failure with preserved ejection fraction, and exercise stress cardiovascular magnetic resonance imaging.
